# Alternative Functions of Cell Cycle-Related and DNA Repair Proteins in Post-mitotic Neurons

**DOI:** 10.3389/fcell.2021.753175

**Published:** 2021-10-20

**Authors:** Remi Akagawa, Yo-ichi Nabeshima, Takeshi Kawauchi

**Affiliations:** ^1^Laboratory of Molecular Life Science, Institute of Biomedical Research and Innovation, Foundation for Biomedical Research and Innovation at Kobe (FBRI), Kobe, Japan; ^2^Department of Physiology, Keio University School of Medicine, Tokyo, Japan

**Keywords:** cell cycle, DNA repair, post-mitotic neurons, neuronal cytoskeleton, extra-cell cycle-regulatory function (EXCERF), cerebral cortical development, G0 phase, expression analysis

## Abstract

Proper regulation of neuronal morphological changes is essential for neuronal migration, maturation, synapse formation, and high-order function. Many cytoplasmic proteins involved in the regulation of neuronal microtubules and the actin cytoskeleton have been identified. In addition, some nuclear proteins have alternative functions in neurons. While cell cycle-related proteins basically control the progression of the cell cycle in the nucleus, some of them have an extra-cell cycle-regulatory function (EXCERF), such as regulating cytoskeletal organization, after exit from the cell cycle. Our expression analyses showed that not only cell cycle regulators, including cyclin A1, cyclin D2, Cdk4/6, p21^cip1^, p27^kip1^, Ink4 family, and RAD21, but also DNA repair proteins, including BRCA2, p53, ATM, ATR, RAD17, MRE11, RAD9, and Hus1, were expressed after neurogenesis, suggesting that these proteins have alternative functions in post-mitotic neurons. In this perspective paper, we discuss the alternative functions of the nuclear proteins in neuronal development, focusing on possible cytoplasmic roles.

## Introduction

Tight regulation of cell cycle progression is fundamental for the development of multicellular organisms, whereas in adulthood, many cells exit from the cell cycle and enter the G0 phase. For example, differentiated neurons never proliferate again throughout their lifetime. In such G0-arrested cells, the expression and/or activity of cell cycle-regulatory proteins should be suppressed. However, increasing evidence indicates that at least some cell cycle proteins, such as cyclin E, p27^Kip1^, and Cdh1, are still expressed after cell cycle exit, and exhibit extra-cell cycle-regulatory functions (EXCERFs) in post-mitotic neurons (Frank and Tsai, 2009; Kawauchi et al., 2013; Kawauchi and Nabeshima, 2019).

**FIGURE 1:**
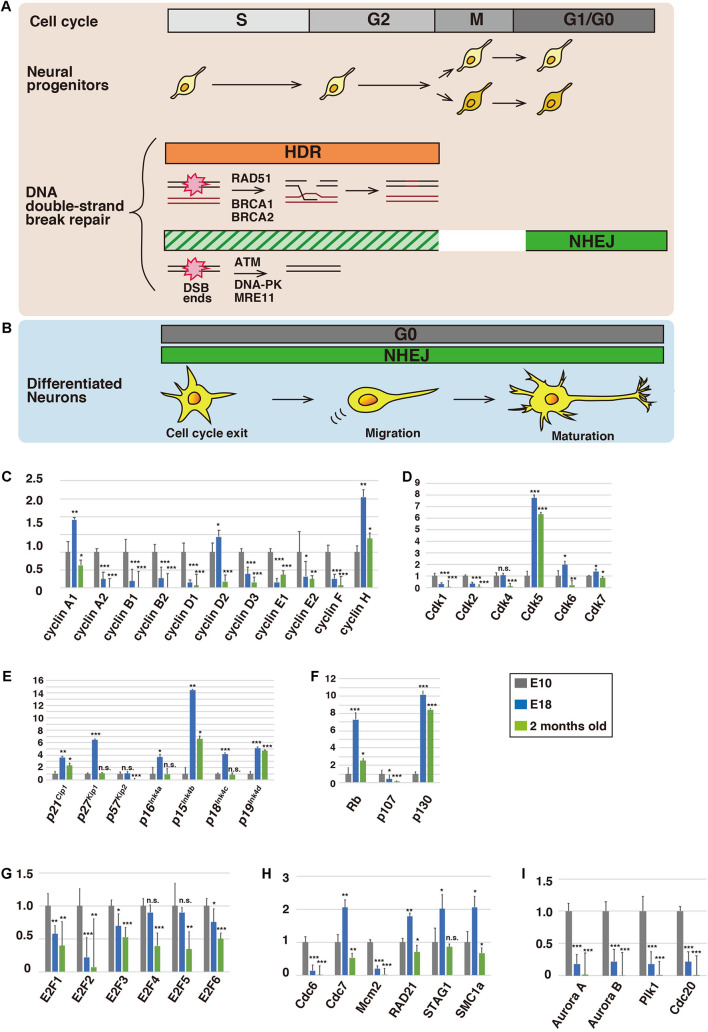
Expression profiles of cell cycle-related proteins in the brain. Schematic illustration of the cell cycle and DNA repair in neural progenitors **(A)** and differentiated neurons **(B)**. During the S and G2 phases, damaged DNA is predominantly repaired via homology-directed repair (HDR), whereas non-homologous end-joining (NHEJ) is utilized at the G1 and G0 phases (see the text for details). **(C–I)** Relative mRNA expression levels of cell cycle-related proteins in cerebral cortices of mice at embryonic day 10 (E10), E18, and 2 months old. Total RNA was extracted from the cerebral cortices of E10, E18, and 2-month-old ICR mice (SLC Japan) using an RNeasy Mini Kit (Qiagen) and transcribed into first-strand cDNA using ReverTra Ace qPCR RT Master Mix (TOYOBO), according to the manufacturer’s instructions. Quantitative real-time PCR was performed with a QuantStudio 3 Real-Time PCR System (Applied Biosystems) using TaqMan Array Fast Plates for cell cycle-related and DNA repair genes (Applied Biosystems) (see also Supplementary Table 1). Relative expression levels were calculated by the comparative Ct (ΔΔCt) method using glyceraldehyde-3-phosphate dehydrogenase (GAPDH) mRNA as an internal control. Animals were handled in accordance with guidelines established by RIKEN-BDR and Institute of Biomedical Research and Innovation, FBRI. Each Y-axis shows the mean ratio ± standard deviation (SD). Significance of differences between the relative mRNA expression levels at E10 and that of E18 or 2 months old was determined by applying Student’s *t* test. *P < 0.05, **P < 0.01, ***P < 0.001, and n.s., no significant difference.

During the cell cycle, DNA is duplicated and equally segregated into two daughter cells. However, DNA is often damaged by endogenous or exogenous sources. If it is misrepaired or left unrepaired, DNA damage, such as DNA double-strand breaks (DSBs), can lead to carcinogenesis or cell death (Mahaney et al., 2009). Checkpoint proteins have crucial roles in the coordination of cell cycle progression with DNA repair. When detecting damaged DNA, checkpoint proteins slow and/or arrest cell cycle progression to allow time for appropriate repair processes (Abraham, 2001). Several types of DNA repair pathways, including NHEJ (non-homologous end-joining) and HDR (homology-directed repair), have been reported (O’Driscoll and Jeggo, 2006). The accuracy of HDR is higher than that of NHEJ, because HDR utilizes homologous DNA sequences. However, homologous DNA sequences are only present during the mid-late S and G2 phases of the cell cycle, and therefore a cell cycle checkpoint predominantly activates HDR during the S and G2 phases for faithful repair (Figure 1A). In contrast, cells at G0/G1 phases utilize the error-prone NHEJ to repair DSBs (Figure 1B; Akagawa et al., 2020). Because at least some DNA repair pathways are active only in proliferating cells, the expression of these proteins should be downregulated at the G0/G1 phases. Considering the EXCERFs of the cell cycle proteins, it seems plausible that some checkpoint and DNA repair proteins may also continue to be expressed and have alternative roles in post-mitotic neurons. However, the expression and functions of these proteins in G0-arrested cells are largely unknown.

In this perspective paper, we describe the expression profiles of the cell cycle, checkpoint, and DNA repair proteins in mouse cerebral cortices at embryonic day 10 (E10) and E18, as well as 2 months old (see figure legends for the experimental procedures). The E10 cerebral cortex mainly consists of proliferating neural progenitors, whereas cortical neurogenesis is completed by E17 (Takahashi et al., 1999). After neurogenesis, astrocyte production (gliogenesis) begins (Hirabayashi and Gotoh, 2005). We confirmed that nestin, a marker for neural progenitors, was expressed at E10 and E18, neurogenic and gliogenic periods, respectively, and that expression of MAP2, a marker for mature neurons, was increased at E18 (Supplementary Figures 1A,B). Expression of glial markers was observed at E18 and/or 2 months old (Supplementary Figure 1C), but previous reports indicate that non-neuronal cells in mouse cerebral cortices account for less than 30% of total cells (Herculano-Houzel, 2014; Keller et al., 2018). In accordance with these findings, the expression of the proliferative marker, Ki67, was reduced at E18 and 2 months old (Supplementary Figure 1A).

Here, we summarize current knowledge about the extra-cell cycle-regulatory functions of cell cycle proteins and discuss possible functions of the cell cycle, checkpoint, and DNA repair proteins in post-mitotic neurons with reference to these expression profiles. We also discuss the cytoplasmic roles of these nuclear proteins, especially focusing on the regulation of the neuronal cytoskeleton.

## Cell Cycle Regulators in Post-Mitotic Neuron

### Cyclins, Cyclin-Dependent Kinases (CDKs), and Cyclin-Dependent Kinase Inhibitor Proteins

Cell cycle progression is controlled by cyclins and CDKs (Morgan, 2008a). Cyclin D forms a complex with Cdk4 or Cdk6, and the cyclin D-Cdk4/6 complex promotes the transition of G1 to S phase. Similarly, cyclin E-Cdk2 and cyclin B-Cdk1 control G1-S transition and M phase progression, respectively. After exit from the cell cycle, CDK activity and expression are generally downregulated. In contrast, Cdk5 is an atypical neuronal CDK that is activated by its specific activators, p35 and p39 (Kawauchi, 2014). Cdk5 is required for various aspects of neural development and function, including neuronal migration, dendrite formation, and synaptic plasticity, through regulation of the neuronal cytoskeleton and membrane trafficking. Indeed, our expression analyses of CDKs indicated that Cdk5 was highly expressed at E18, when neurogenesis is completed (Figure 1D).

To our surprise, some cell cycle-related conventional CDKs were also expressed after neurogenesis. Expression of Cdk4 was maintained at E18, while expression of Cdk6 and Cdk7 was increased at E18, compared with E10 (Figure 1D). Furthermore, the expression of cyclin D2 and cyclin H, binding partners of Cdk4/6 and Cdk7, respectively, was also increased at E18 (Figure 1C). Considering that cyclin H and Cdk7 are components of Cdk-activating kinase (CAK) and activate Cdk4/6 (Schachter et al., 2013), the CAK and cyclin D2-Cdk4/6 cascade may have a functional role in post-mitotic neurons.

While cyclin E1 expression was decreased at E18, it was slightly increased again in 2-month-old animals (Figure 1C). A previous report indicates that overexpression of a fusion protein of truncated cyclin E and Cdk2 leads to M-phase entry in differentiated primary neurons (Walton et al., 2019), although cell cycle reentry may result in neuronal cell death (Barrio-Alonso et al., 2018). However, Cdk2 was barely expressed at 2 months old (Figure 1D), suggesting that cyclin E has a Cdk-independent function (Chu et al., 2021). Consistent with this, it has been reported that cyclin E is involved in synaptic plasticity through the negative regulation and sequestration of Cdk5, suggesting that cyclin E functions without activation of CDKs (Odajima et al., 2011). Our expression analyses showed that the expression of cyclin A1 was increased at E18, but its binding partners, Cdk1 and Cdk2, exhibited low expression levels (Figures 1C,D). Cyclin A1 may also play a role independent of the activation of CDKs in post-mitotic neurons, similar to cyclin E.

CDK inhibitor proteins (CKI) negatively regulate the activities of CDKs (Sherr and Roberts, 1999; Morgan, 2008b). CKIs are classified into the cip/kip and ink4 families. Among the cip/kip family proteins, p21^Cip1^, p27^Kip1^, and p57^Kip2^, p27^Kip1^ showed dramatically increased expression at E18 (Figure 1E). Previous studies indicate that p27^Kip1^ plays crucial roles in neuronal migration and morphological changes via suppression of a small GTPase, RhoA, leading to activation of an actin-binding protein, cofilin (Kawauchi et al., 2006; Nishimura et al., 2014). p27^Kip1^ also controls microtubule polymerization and microtubule-dependent axonal transport (Godin et al., 2012; Morelli et al., 2018). Both p21^Cip1^ and p57^Kip2^ are reported to be involved in cofilin-mediated actin organization in non-neuronal cells (Frank and Tsai, 2009), and p57^Kip2^ is required for neuronal migration *in vivo* (Itoh et al., 2007). Thus, the cip/kip family proteins exhibit extra-cell cycle regulatory functions in post-mitotic neurons.

In contrast, it is unclear whether the ink4 family proteins acquire extra-cell cycle regulatory functions. Double knockout of p27^Kip1^ and p19^Ink4d^ results in postnatal neuronal proliferation in the cerebral cortex (Zindy et al., 1999), suggesting that p19^Ink4d^ suppresses cell cycle progression, in cooperation with p27^Kip1^. Our expression analyses showed that expression of all ink4 family proteins, p16^Ink4a^, p15^Ink4b^, p18^Ink4c^, and p19^Ink4d^, was increased at E18 (Figure 1E). Unlike cip/kip family members, the ink4 family proteins only suppress the activity of cyclin D-Cdk4/6. Considering that cyclin D2 and Cdk4/6 are expressed at E18, the ink4 family proteins may have an additional function besides the suppression of cell cycle reentry.

### Retinoblastoma and E2F Family Members (Regulators at G1-S)

Major substrates of cyclin D-Cdk4/6 and cyclin E-Cdk2 are retinoblastoma (Rb) family proteins, Rb (retinoblastoma 1), p107 (retinoblastoma-like 1), and p130 (retinoblastoma-like 2) (Trimarchi and Lees, 2002). The Rb proteins bind to and suppress the transcriptional activity of E2F1, E2F2, and E2F3a. The CDK-mediated phosphorylation of Rb releases the E2Fs from Rb, enabling them to activate the transcription of genes involved in the G1-S transition. The E2Fs undergo proteasome-dependent degradation mediated by cyclin F, an E3 ubiquitin ligase, during the late S and G2 phases (Clijsters et al., 2019). While the Rb proteins and the activator E2Fs are involved in the G1-S transition, we found that E2F1, E2F3, Rb, and p130 were still expressed after neurogenesis (Figures 1F,G). Rb and E2F3 are reported to regulate neuronal migration in the developing cerebral cortex, in addition to their roles in the proliferation of neural progenitors. Because the expression levels of Rb and p130 were increased at E18 and 2 months old, these proteins may have additional functions in mature neurons. Indeed, Rb is reported to be required for neuronal survival (Andrusiak et al., 2012).

In contrast to the activator E2Fs, E2F4, and E2F5 act as transcriptional repressors and promote cell cycle exit (Trimarchi and Lees, 2002). In accordance with this, our data showed that expression of both E2F4 and E2F5 was maintained after neurogenesis (Figure 1G). E2F6 is reported to suppress cell cycle-related genes, including cyclin B1, during neuronal differentiation, and its expression was also observed at E18 and 2 months old (Figure 1G).

### Pre-replicative Complex (Regulators at S Phase)

In dividing cells, DNA replication occurs at the S phase and the replicated sister chromatids are held together by a cohesin complex from the S phase until anaphase (Morales and Losada, 2018). Cdc6, an AAA+ ATPase protein, loads an MCM (mini-chromosome maintenance) helicase protein complex on double-stranded DNA to form a pre-replicative complex (pre-RC) at replicative origins (Fragkos et al., 2015). Consistent with their roles in DNA replication, Cdc6 and Mcm2, a component of the MCM complex, were expressed at E10, when neural progenitors actively proliferate, but the expression levels were reduced at E18 and 2 months old (Figure 1H). However, the expression of Cdc7, an upstream kinase of MCM2 (Chuang et al., 2009), was increased at E18 (Figure 1H). In addition, the expression profiles of RAD21, STAG1, and SMC1a, components of the cohesin complex, are similar to each other (Figure 1H). These data suggest unidentified functions of Cdc7 and the cohesin complex in post-mitotic neurons.

### Aurora Kinases and Polo-Like Kinase-1 (Regulators at M Phase)

In addition to cyclin B-Cdk1, Aurora kinases and Polo-like kinase-1 (Plk1) play critical roles in cell division (Joukov and De Nicolo, 2018). Aurora A is accumulated around the centrosomes and regulates centrosome maturation and separation, whereas Aurora B, a component of chromosomal passenger complex (CPC), is concentrated at inner centromeres and regulates attachments between kinetochores and spindle microtubules during mitosis. Both Aurora A and Aurora B phosphorylate Plk1, which is colocalized with Aurora A at the centrosomes and with Aurora B at the centromeres and central spindles. Plk1 regulates centrosome maturation and separation, as well as the attachments between kinetochores and spindle microtubules. Interestingly, these mitotic kinases showed similar expression profiles at E10, E18, and 2 months old (Figure 1I), implying that the Aurora kinases-Plk1 axis may be preserved in post-mitotic neurons. Because Aurora A is reported to control neurite elongation and neuronal migration (Mori et al., 2009; Takitoh et al., 2012), Aurora B and Plk1 may also be involved in these neuronal maturation events possibly through the regulation of microtubule organization or primary cilia dynamics, both of which are related to Aurora A function at least in part (Bertolin and Tramier, 2020).

Aurora kinases are degraded by an APC/C ubiquitin ligase complex (Lindon et al., 2016). APC/C is activated by binding to Cdh1 or Cdc20, both of which are shown to regulate neuronal morphologies (Yang et al., 2010).

## Checkpoint and DNA Repair Proteins in Post-Mitotic Neurons

### RAD51, BRCA1 and BRCA2

**FIGURE 2:**
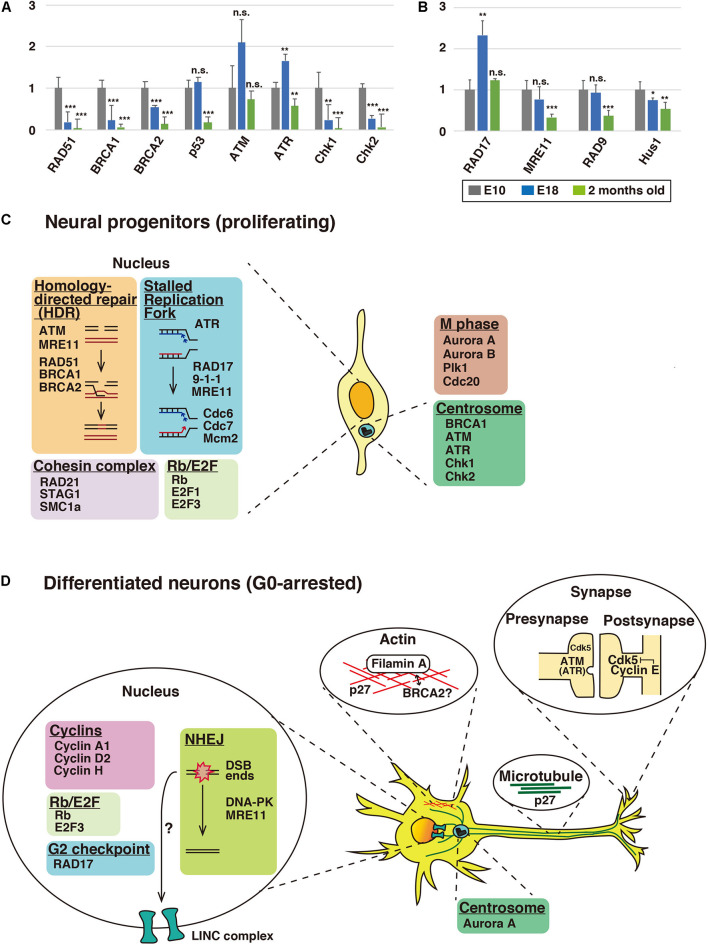
Expression profiles of cell cycle checkpoint and DNA repair proteins in the brain. **(A,B)** Relative mRNA expression levels of checkpoint and DNA repair proteins in cerebral cortices of mice at E10, E18, and 2 months old. See the legend of Figure 1 for the quantitative real-time PCR method. Each *Y*-axis shows the mean ratio ± standard deviation (SD). Significance of differences between E10 and E18 or 2 months old was determined by applying Student’s *t* test. *P < 0.05, **P < 0.01, ***P < 0.001, and n.s., no significant difference. Schematic illustration depicting alternative functions of DNA repair and cell cycle proteins in neural progenitors **(C)** and differentiated neurons **(D)**. In proliferating neural progenitors, cell cycle and DNA repair proteins basically function in the nucleus. In differentiated neurons, some of these nuclear proteins, such as p27^Kip1^ and ATM, relocate to the cytoplasm and show extra-cell cycle/DNA repair-regulatory functions, including the regulation of neuronal cytoskeleton, in post-mitotic neurons. While p27^Kip1^ is associated with neuronal cytoskeleton, ATM and ATR control clathrin-mediated endocytosis in excitatory and inhibitory synapses, respectively. Centrosomal Aurora A and synaptic cyclin E are reported to control neuronal migration and synapse plasticity, respectively. BRCA2 may interact with Filamin A. It is also possible that DNA double-strand breaks may have a role in cytoskeletal regulation via LINC (linkers of the nucleoskeleton to the cytoskeleton) complex (see the text for details).

Homology-directed repair (HDR) is active only in the S/G2 phases and repairs DNA double-strand breaks (DSBs) using homologous DNA sequences. RAD51 forms a right-handed helical nucleoprotein filament on single-stranded DNA through interaction with BRCA1 (breast cancer susceptibility gene 1), a tumor suppressor gene product that was originally identified in hereditary breast and ovarian cancer (Scully et al., 1997), and the related molecule, BRCA2 (Chen et al., 1999; Davies et al., 2001; Prakash et al., 2015). Our expression analyses showed that RAD51 expression was decreased at E18 and 2 months old (Figure 2A). In spite of the low expression level in adult brains, mutation in the *RAD51* gene causes a congenital mirror movements disorder (Depienne et al., 2012), suggesting that local expression of RAD51 may be important for the higher brain function.

The expression of BRCA1 was observed at E10 (Figure 2A). Conditional gene ablation of BRCA1 in neural progenitors in the developing cerebral cortex by using Emx1-Cre results in a reduction of cortical thickness, though a six-layered cortical structure is preserved (Pulvers and Huttner, 2009). In the conditional knockout mice, the large exon 11 of the *BRCA1* gene is deleted, resulting in an in-frame fusion of truncated *BRCA1* genes. In contrast, *BRCA1* null deletion in neural progenitors results in severe disruption of the upper cortical layer neurons (Pao et al., 2014). The layer 6 and a part of layer 5 neurons are still observed but the polarity of these neurons is disturbed. In the absence of BRCA1, a massive apoptosis occurs in the neural progenitors, but this does not occur in BRCA1/p53 double-knockout mice, suggesting that BRCA1 depletion induces p53-dependent apoptosis in the neural progenitors. However, the neuronal polarity defects are not rescued in the double-knockout mice. It is unclear whether the p53-independent regulation of neuronal polarity is associated with the DNA repair function of BRCA1. Considering that BRCA1 is localized at the centrosome, as well as damaged DNA (Mullee and Morrison, 2016), it is possible that the neuronal polarity is mediated by the centrosomal BRCA1, because the centrosome and centrosomal microtubules play important roles in neuronal polarity formation (De Anda et al., 2005).

Loss of BRCA2 in neural progenitors leads to microcephaly, similar to that seen in BRCA1-knockout mice (Frappart et al., 2007). In the absence of BRCA2, p53-dependent apoptosis occurs in the neural progenitors of the cerebellar granule neurons, possibly due to defects in the DNA repair function of BRCA2. It has also been reported that BRCA2 localizes on the midbody during cytokinesis in non-neuronal cells, and loss of function of BRCA2 leads to a defect in cytokinesis. The midbody is a microtubule- and actin-rich structure, and BRCA2 preferentially binds to filamin-A, an actin-binding protein, which recruits BRCA2 to the midbody (Jingyin et al., 2009; Mondal et al., 2012). Interestingly, mutation in the human *filamin-A* gene causes periventricular heterotopia (PVH) with neuronal migration defects (Fox et al., 1998). Furthermore, moderate expression of BRCA2 was observed after the neurogenic period, whereas BRCA1 expression was reduced at E18 (Figure 2A). Thus, BRCA2 may play a role in the differentiated neurons at later developmental stages, in cooperation with filamin A.

### ATM, ATR, and DNA-PK

The PI3K-related kinase (PIKK) family consists of ATM (ataxia-telangiectasia mutated), ATR (ATM and RAD3-related kinase), and DNA-PKcs (DNA-dependent protein kinase catalytic subunit), which play essential roles in the DNA damage response (Falck et al., 2005). ATM is a key regulator of a DNA double-strand break repair and cell-cycle checkpoint, whereas ATR is involved in a DNA single-strand break repair, as well as cell-cycle checkpoint regulation (Abraham, 2001; Maréchal and Zou, 2013). In contrast to BRCA1 and BRCA2, ATM expression was still observed at 2 months old (Figure 2A). Consistent with this, ATM-deficient mice are reported to exhibit a reduction of long-term potentiation (LTP) in the hippocampus, suggesting that ATM is involved in synaptic plasticity in adult brain (Li et al., 2009).

Previous studies have shown that ATR contributes to the proliferation of neural progenitors (Zhou et al., 2012). ATR expression was increased at E18 (Figure 2A), and ATR is reported to regulate the migration of post-mitotic neurons (Kidiyoor et al., 2020). ATR was also expressed in the cerebral cortex of 2-month-old mice, although the expression level was decreased compared to E18 brains (Figure 2A). In contrast, the expression of Chk2 and Chk1, major substrates of ATM and ATR, respectively, disappeared at 2 months old, suggesting that other substrates may be involved at this time point (Figure 2A). Both ATR and ATM are reported to interact with clathrin and AP-2 clathrin adaptor complex and to regulate the endocytic recycling of synaptic vesicles (Cheng et al., 2017). The same report also suggested that ATR functions at the synapse of inhibitory neurons, whereas ATM functions at the synapse of excitatory neurons.

While the cytoplasmic function of DNA repair proteins was reported as described above, several other studies indicate that DNA double-strand break (DSB) ends also indirectly interacts with the cytoskeleton. Microtubules and kinesins, plus-end directed motor proteins, control the mobility of the DSB ends through LINC (linkers of the nucleoskeleton to the cytoskeleton) complex (Lottersberger et al., 2015). LINC complex is known to physically connect lamins, intermediate filaments that lie just beneath the inner nuclear membrane, with microtubules (Méjat and Misteli, 2010). However, when DSBs occur, LINC complex mediates kinesin-dependent mobility of the DSB ends in the nucleus, which increases the efficiency of ligation of the DSB ends during non-homologous end-joining (NHEJ) (Lottersberger et al., 2015). Thus, LINC complex links damaged DNA in the nucleus with microtubules in the cytoplasm. Interestingly, double knockout mice of Sun1 and Sun2 or Nesprin-1 and -2 (components of LINC complex) exhibit neuronal migration defects in the cerebral cortex (Zhang et al., 2009), implying that the interaction between nuclear DNA and the neuronal cytoskeleton may play a role in neural development.

In addition to the microtubule-dependent movement of the DSB ends, DSB-dependent microtubule regulation has been reported. Ionizing radiation-induced DSBs promote centrosome-dependent microtubule nucleation capacity and microtubule polymerization, and this phenomenon is referred as to the DSB-induced microtubule dynamics stress response (DMSR) (Ma et al., 2021). Treatment with an inhibitor of DNA-PK, a crucial regulator of the NHEJ, but not ATM or ATR, impairs DMSR, suggesting that DNA-PK-mediated NHEJ in the nucleus is involved in the regulation of microtubule dynamics. DNA-PKcs, a catalytic subunit of DNA-PK, promotes the phosphorylation of Akt at Thr308 at the centrosome, and suppression of the Akt activity attenuates DMSR. Because DMSR occurs only at G0 and G1 phases (Ma et al., 2021), neurons may utilize a similar pathway to regulate microtubule dynamics.

### G2 Checkpoint Proteins

Upon DNA damage in the G2 phase, ATR phosphorylates RAD17, a component of replication factor C (RFC)-related checkpoint clamp loader complex, which recruits the 9-1-1 (RAD9-Hus1-RAD1) complex and the MRE11-RAD50-NBS1 (MRN) complex to sites of DNA damage (Bermudez et al., 2003; Wang et al., 2006, 2014; Yang and Zou, 2006). However, RAD17 expression was significantly increased at E18, when most cells have exited from the cell cycle (Figure 2B). Furthermore, the expression of RAD9, Hus1, and MRE11, both of which are recruited by RAD17, was also observed at E18, suggesting that these G2 DNA damage checkpoint proteins may play a role in the G0 phase (Figure 2B). Consistent with this notion, phosphorylated RAD17 is present in the nuclei of cells in mouse adult brains and the cytoplasm of some spinal motor neurons (Martin and Wong, 2017).

## Conclusion and Future Directions

In our expression analyses, we found that several cell cycle and DNA repair genes are expressed at E18 and 2 months old, when cortical neurogenesis has already been completed. DNA damage response presumably still occurs in post-mitotic neurons, but several types of DNA repair, such as HDR, are expected to be inactive after neurogenesis. To identify the putative alternative functions of the DNA repair proteins, more detailed analyses, including subcellular localizations, domain functions, and interacting proteins, will be needed.

Herein, we propose that radiation-sensitive mutant (rad) genes such as *RAD17* and *RAD9* contribute to some neuronal functions, based on the fact that they are expressed at E18 and 2 months old. Historically, rad mutants in yeast were identified after UV-irradiation, which not only induces DNA damage but also affects the activity of various proteins, such as JNK, independently of DNA damage (Adler et al., 1995). In addition, low doses of γ-irradiation rapidly alter potassium channel activity (Kuo et al., 1993). These findings suggest that exposure to UV or γ-irradiation not only causes DNA damage but also disturbs cytoplasmic homeostasis.

Our present study indicates that many cell cycle proteins show post-mitotic expression beyond expectation, which may contribute to further development of the field of EXCERFs (extra-cell cycle regulatory functions) of cell cycle proteins. This may also be the case for certain DNA repair proteins, whose alternative functions have barely been examined (Figures 2C,D). Neurons are non-cycling G0-arrested cells and may be good models for studies of these putative additional functions.

## Data Availability Statement

The raw data supporting the conclusions of this article will be made available by the authors, without undue reservation.

## Ethics Statement

The animal study was reviewed and approved by RIKEN-BDR and Institute of Biomedical Research and Innovation, FBRI.

## Author Contributions

TK and RA conceived the project, designed the experiments, and wrote the manuscript. TK supervised the project. RA performed the experiments and conducted the data analysis. Y-iN administrated the experimental environments and provided helpful comments. All authors contributed to the article and approved the submitted version.

## Conflict of Interest

The authors declare that the research was conducted in the absence of any commercial or financial relationships that could be construed as a potential conflict of interest.

## Publisher’s Note

All claims expressed in this article are solely those of the authors and do not necessarily represent those of their affiliated organizations, or those of the publisher, the editors and the reviewers. Any product that may be evaluated in this article, or claim that may be made by its manufacturer, is not guaranteed or endorsed by the publisher.
