# Supplementary figures and images for: Alternative Functions of Cell Cycle-Related and DNA Repair Proteins in Post-mitotic Neurons
Source: Front Cell Dev Biol. 2021 Oct 20;9:753175. doi: 10.3389/fcell.2021.753175 (PMC8564117; doi:10.3389/fcell.2021.753175)

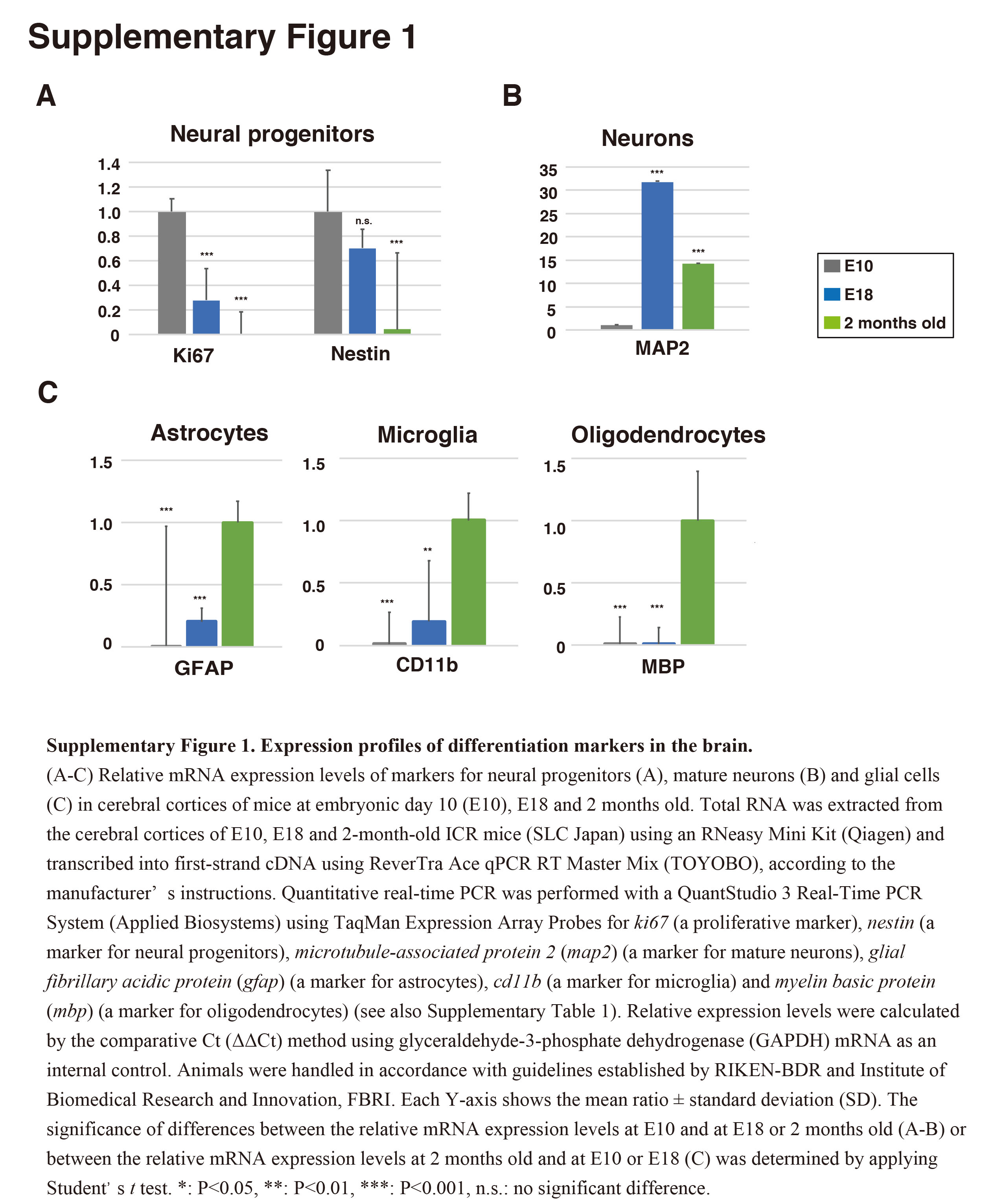

Supplement: Supplementary file 1 [file Image_1.jpg]
