# Supplementary material for: Alternative Functions of Cell Cycle-Related and DNA Repair Proteins in Post-mitotic Neurons
Source: Front Cell Dev Biol. 2021 Oct 20;9:753175. doi: 10.3389/fcell.2021.753175 (PMC8564117; doi:10.3389/fcell.2021.753175)
Supplement: Supplementary file 2 [file Table_1.DOCX]

Supplementary Material

**Supplementary Table 1.** The assay ID numbers of TaqMan Array Fast Plates for cell cycle-related and DNA repair genes.

|  | Target name | assay ID |
| --- | --- | --- |
| Figure 1C | cyclin A1 | Mm00432337_m1 |
|  | cyclin A2 | Mm00438063_m1 |
|  | cyclin B1 | Mm03053893_gH |
|  | cyclin B2 | Mm01171453_m1 |
|  | cyclin D1 | Mm00432359_m1 |
|  | cyclin D2 | Mm00438070_m1 |
|  | cyclin D3 | Mm01612362_m1 |
|  | cyclin E1 | Mm00432367_m1 |
|  | cyclin E2 | Mm00438077_m1 |
|  | cyclin F | Mm00432385_m1 |
|  | cyclin H | Mm00445740_m1 |
| Figure 1D | Cdk1 | Mm00772472_m1 |
|  | Cdk2 | Mm00443947_m1 |
|  | Cdk4 | Mm00726334_s1 |
|  | Cdk5 | Mm00432447_g1 |
|  | Cdk6 | Mm01311342_m1 |
|  | Cdk7 | Mm00802284_m1 |
| Figure 1E | p21 (Cip1) | Mm04207341_m1 |
|  | p27 (Kip1) | Mm00438168_m1 |
|  | p57 (Kip2) | Mm00438170_m1 |
|  | p16 (Ink4a) | Mm00494449_m1 |
|  | p15 (Ink4b) | Mm00483241_m1 |
|  | p18 (Ink4c) | Mm00483243_m1 |
|  | p19 (Ink4d) | Mm00486943_m1 |
| Figure 1F | Rb | Mm00485586_m1 |
|  | p107 | Mm01250721_m1 |
|  | p130 | Mm01242468_m1 |
| Figure 1G | E2F1 | Mm00432936_m1 |
|  | E2F2 | Mm00624964_m1 |
|  | E2F3 | Mm01138833_m1 |
|  | E2F4 | Mm00514160_m1 |
|  | E2F5 | Mm00468171_m1 |
|  | E2F6 | Mm00519030_m1 |
| Figure 1H | Cdc6 | Mm03048221_m1 |
|  | Cdc7 | Mm00438122_m1 |
|  | Mcm2 | Mm00484815_m1 |
|  | RAD21 | Mm00485474_m1 |
|  | STAG1 | Mm01224921_m1 |
|  | SMC1a | Mm00490624_m1 |
| Figure 1I | Aurora A | Mm01248177_m1 |
|  | Aurora B | Mm01718146_g1 |
|  | Plk1 | Mm00440924_g1 |
|  | Cdc20 | Mm00650983_g1 |
| Figure 2A | RAD51 | Mm00487905_m1 |
|  | BRCA1 | Mm01249840_m1 |
|  | BRCA2 | Mm01218747_m1 |
|  | p53 | Mm01731290_g1 |
|  | ATM | Mm01177457_m1 |
|  | ATR | Mm01223626_m1 |
|  | Chk1 | Mm01176757_m1 |
|  | Chk2 | Mm00443844_m1 |
| Figure 2B | RAD17 | Mm01288359_m1 |
|  | MRE11 | Mm00450600_m1 |
|  | RAD9 | Mm00487923_m1 |
|  | Hus1 | Mm00476200_m1 |
| Control | GAPDH | Mm99999915_g1 |
| Supplementary Figure 1A | Ki67 | Mm01278617_m1 |
|  | Nestin | Mm00450205_m1 |
| Supplementary Figure 1B | MAP2 | Mm00485230_m1 |
| Supplementary Figure 1C | GFAP | Mm01253033_m1 |
|  | CD11b | Mm00434455_m1 |
|  | MBP | Mm01266402_m1 |
